# The impact of age and sex on healthcare expenditure of households in Bangladesh

**DOI:** 10.1186/2193-1801-3-435

**Published:** 2014-08-14

**Authors:** Abdur Razzaque Sarker, Rashidul Alam Mahumud, Marufa Sultana, Sayem Ahmed, Wahid Ahmed, Jahangir AM Khan

**Affiliations:** Health Economics and Financing Research Group, Center for Equity and Health Systems, International Centre for Diarrhoeal Disease Research, Bangladesh (icddr,b), Dhaka, Bangladesh; Centre for Excellence in Universal Health Coverage at icddr,b and James P Grant School of Public Health, BRAC University, Dhaka, Bangladesh; Adjunct Faculty, Health Economics Unit, Department of Learning, Informatics, Management and Ethics, Karolinska Institutet, Stockholm, Sweden

**Keywords:** Health care expenditure, Reproductive age, Sex, Bangladesh

## Abstract

The impact of age and sex on health care expenditure has recently become one of the major concerns in many developing countries like Bangladesh. Age and sex differences in the use of health care services can be substantial at several stages of life which are reflected in overall healthcare expenditure.

We examined the impact of age and sex of the population on overall healthcare expenditure of households in Bangladesh.

A total of 10,705 populations who spent for receiving any type of healthcare services were analyzed from Bangladesh Household Income and Expenditure Survey data, 2010. Sex and age group were considered as childhood (0–19), young adult (20–39), middle-aged adult (40–64), senior aged (65–84) and old senior aged (84+) for the entire analysis. Total healthcare expenditure was derived by considering direct cost such as physician’s fee, cost of medicine, diagnostic, transportation, tips and informal payment etc. Indirect and intangible cost was not considered in the analysis.

The study found that overall health care expenditure of male (US$ 11.5) is higher than female (US$ 11.2) while this is higher for female (US$ 14.2) than male (US$ 11.3) in the reproductive age. The highest health expenditure was observed in male (US$ 69.7) of age 65–69 years and in female (US$ 23.4) of age 75–79 years. The cost for hospitalization was significantly higher (US $23.7) for female than male (US$ 21.1). Overall health expenditure was observed to be significantly higher in elderly than younger people.

These findings provide an experimental framework for the continuing inquiry of equity in the allocation of health care expenditure between male and female at different age, which suggest current health care system in Bangladesh place a significant financial strain on the elderly population.

## Introduction

The impact of age and sex on health care expenditure has recently become one of the major concerns in many developing countries like Bangladesh (Meng and Yeo 2005). According to latest health bulletin of ministry of health and family welfare of Bangladesh, the estimated total population was 154.8 million (MOHFW 2013). The demographic structure is changing faster and the population pyramid is wider at the bottom than the top and narrows slightly at the youngest age group (BDHS 2011). The life expectancy at birth for both sexes is increasing from 65 to 69 years according to latest health bulletin (MOHFW 2013). This demographic nature have attracted considerable attention from policy makers, healthcare managerial level and public health expertise since it will exert on rising healthcare costs. However, it is also important that the age and sex specific utilization pattern varies among the different age groups over time. The utilization of health care services normally depends on particular health problem that reflected in overall healthcare expenditure. Health care expenditure is strongly age dependent, an experience that takes on increasing significance as the childhood generation ages consequently faster change of ageing (Alemayehu and Warner 2004). Also the health care expenditures are lowest for children after the first year of life, rise slowly throughout adult life, and increase exponentially after 50 years age (Meerding et al. 1998). In earlier study found that the health care expenditures for the elderly people are about four to five times in their early teens and the senior oldest group (85+) consumes three times as much health care per person as those 65–74, and twice as much as those 75–84 (Bradford and Max 1996; Fuchs 1998).

Previous studies examined that age of the population strongly influenced the health care expenditures (Gerdtham et al. 1992; Khan et al. 2004), and also established the relationship between age and health care expenditure. However, the age–specific health care utilization changes over the different age groups (Seshamani and Gray 2002). Further, other studies reported that health care utilization was more in female than male (Hibbard and Pope 1983; Verbrugge and Wingard 1987; Waldron 1983). In general, female have a tendency to use preventive and diagnostic services more frequently than male, but the emergency services utilization is more in male Gómez (2002). The Goodman’s model found that age effect the individual’s health care expenditure, where health was considered as human capital that depreciates with age (Grossman 1972). However, it was also examined that aged people invest more to support the health resources supply (Folland et al. 2003). Again, few studies suggested that clinicians treat younger patients differently than they treat older patients, adjusting for disease severity and also for patient preferences (James et al. 2008; Schwarzkopf et al. 2012; Leena et al. 2007). The objective of the study was to focus whether and to what extent age and sex of the population have impact on overall healthcare expenditure of households in Bangladesh. Since this paper focused mainly on healthcare expenditure attributed for individuals, and hence, was based on household level data.

## Methods

The present study derived from Household Income and Expenditure Survey-2010. A total of 10,705 populations who spent for receiving any healthcare services were analyzed and thus who didn’t spend on healthcare services was excluded (HIES 2011). Like earlier study, sex and age group were considered as childhood (0–19), young adult (20–39), middle-aged adult (40–64), senior aged (65–84) and old senior aged (84+) for the entire analysis (Alemayehu and Warner 2004). The sampling technique, survey design, survey instruments, measuring system and quality control have been described elsewhere (HIES 2011). Total healthcare expenditure was derived by summing up direct medical cost and direct non-medical cost. Direct medical costs included hospital outpatient fees, medicines, admission or registration fees, physician fees, diagnostic fees, and any other associated medical supplies. The direct non-medical costs include transportation and conveyance, lodging, tips and other associated costs. Indirect costs like income or productivity losses were not captured in this study. However, like other study, intangible costs that is the costs related to suffering and grief, were also excluded from this analysis (Sarker et al. 2013). Data were entered into Microsoft Excel 2007 and statistical analysis was performed using STATA-12. ANOVA was performed to examine the health care expenditure among the human life stages. Results were presented as an average, standard deviations with mean differences, in US$ applying the exchange rate (US$ 1 = 69 BDT) during the data collection year (2010). To estimate the relationship of healthcare expenditure (HE) to age and sex, the following interactive model was used as suggested earlier study (Diehr et al. 1999).


## Results

**Figure 1:**
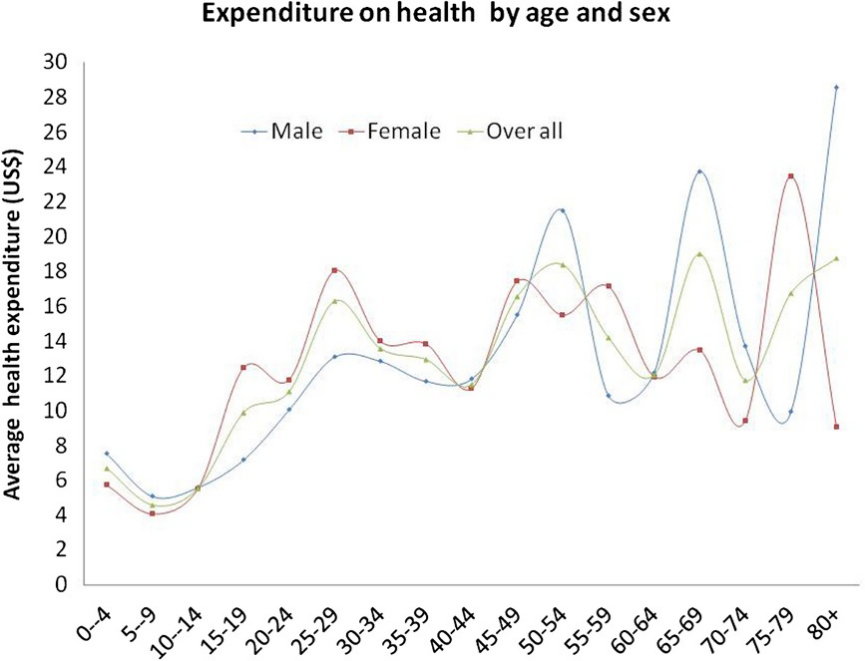
**Age and sex specific health expenditure curve (HEC).**

A total of 10,705 population including male (47.05%) and female (52.95%) were enrolled in this study. The highest health expenditure was observed in case of male (US$ 69.7) with 65–69 years of age and in case of female (US$ 23.4) with 75–79 years of age (Table 1). However, healthcare expenditure was higher (US$ 14.1) in female than man (US$ 11.7) in the reproductive period (Figure 1). Considering the under five age group, healthcare spending was significantly higher in male child (US$7.5) compared to female (US$ 5.7). The overall health care expenditure of male (US$ 11.5) was consistently higher than female (US$ 11.2).

**Table 1:** **Household average health expenditure in US$ (mean ± SD) across age groups**

Age	Male	Female	Mean difference (95% CI of mean difference)
Up to 4	7.52 ± 35.66	5.72 ± 16.74	1.80** (0.78-2.54)
5-9	5.06 ± 10.31	4.05 ± 8.25	1.01* (0.87-1.98)
10-14	5.57 ± 16.04	5.51 ± 21.15	0.06 (-0.05-0.59)
15-19	7.16 ± 11.84	12.45 ± 40.91	-5.29*** (-7.87-0.076)
20-24	10.03 ± 20.40	11.77 ± 26.93	-1.74* (-2.54-1.50)
25-29	13.07 ± 33.90	18.05 ± 104.97	-4.98*** (-5.65-0.05)
30-34	12.82 ± 30.35	13.98 ± 39.91	-1.16* (-2.13-0.87)
35-39	11.66 ± 37.63	13.83 ± 37.90	-2.16** (-3.42-1.54)
40-44	11.80 ± 23.45	11.26 ± 28.39	0.54 (0.041-1.25)
45-49	15.49 ± 54.78	17.43 ± 68.05	-1.94* (-2.54-2.65)
50-54	21.47 ± 81.42	15.48 ± 36.28	5.99** (1.43-7.54)
55-59	10.83 ± 17.24	17.13 ± 38.98	-6.31*** (-7.86-1.04)
60-64	12.14 ± 23.93	11.94 ± 19.24	0.20 (-0.32-0.76)
65-69	69.72 ± 651.40	13.47 ± 40.73	56.25*** (45.65-67.98)
70-74	13.68 ± 25.95	9.42 ± 11.93	4.26** (3.76-6.98)
75-79	9.91 ± 16.70	23.47 ± 102.80	-13.56*** (-15.98-(-10.76))
80+	28.57 ± 77.51	9.08 ± 13.60	19.49*** (13.87-26.98)
Total	11.51 ± 108.12	11.20 ± 44.85	0.30 (-0.05-1.23)

NB: ***, ** and * denote 1%, 5% and 10% significance level respectively, 1 US$ = 69 BDT in 2010.

### Healthcare expenditure across human life stages

In healthcare expenditure across human life stage was presented in Table 2. The average healthcare expenditure was higher for male at the senior aged (US$ 34.9) followed by old senior age (US$ 30.6). However, in female, the highest healthcare expenditure was observed in middle aged adult (US$ 14.6) followed by young adult (US$ 14.5). ANOVA result showed significant difference health care expenditures for all the human life stages (P < 0.00).

**Table 2:** **Household average healthcare expenditure in US$ (mean ± SD) across human life stage**

Human life stage	Male	Female	Mean diff (95% CI)	F-value
Childhood (0–19 years)	6.40 ± 24.20	6.33 ± 22.30	0.07 (-0.03-1.01)	10.67***
Young adult (20–39 years)	11.87 ± 31.47	14.53 ± 62.62	-2.8* (-3.21-0.056)	
Middle-aged adult (40–64 years)	14.60 ± 48.81	14.67 ± 43.86	-0.07 (-1.26 -0.098)	
Senior aged (65 – 84 years)	34.90 ± 392.52	13.09 ± 51.98	21.81*** (15.87-24.87)	
Old senior aged (85 and above)	30.59 ± 87.65	12.52 ± 16.64	18.07*** (16.45-23.65)	
Total	11.51 ± 108.12	11.20 ± 44.85	0.31 (0.051-0.986)	

NB: *** and * denote 1% and 10% significance level respectively.

### Distribution of healthcare expenditure considering sex

The distribution of healthcare expenditure considering sex revealed that medicine cost (US$ 7.51) was the highest cost driver followed by diagnostic test (US$ 1.16) in case of male (Table 3). Similarly, in case of female, medicine cost (US$ 6.45) was also the highest cost driver. However, in case of tips, which considered often as informal payment was relatively higher in female (US$ 0.29) than male (US$ 0.06) which also depicted the higher utilization of maternity services by female.

**Table 3:** **Distribution of average healthcare expenditure in US$ by sex**

Particulates	Male	Female	Mean diff (95% CI of mean diff)
Physician fee	0.77	0.8	-0.03 (-0.67 to 0.75)
Hospital cost	0.79	0.8	-0.01 (-0.72 to 0.54)
Medicine cost	7.51	6.45	1.06** (0.43 to 1.89)
Diagnostic test	1.16	1.39	-0.23 (-0.89 to 0.43)
Conveyance cost	0.72	0.63	0.09 (-0.10 to 0.28)
Tips cost	0.06	0.29	-0.23 (-1.47 to 1.01)
Other costs	0.5	0.39	0.11 (-0.17 to 0.39)
Maternity clinic cost	—	0.23	—
Maternity midwife cost	—	0.03	—
Maternity others	—	0.21	—
Total	11.51	11.2	0.30 (-0.06 to 0.66)

NB: ** denote 5% significance level.

### Relationship of healthcare expenditure to age and sex

Table 4 showed the relationship between healthcare expenditure on age and sex. Healthcare expenditure is significantly associated with age, which means that healthcare expenditures were clearly age dependent; an aging population will imply increasing total healthcare expenditures. However, no such relation was established considering sex.

**Table 4:** **Relationship of healthcare expenditure in US$ to age and sex**

Variables	Coefficients	Std. err.	(95% CI)	
			Lower	Upper
Constant	1.53***	3.96	-6.24	9.29
Age	0.40***	0.11	0.17	0.62
Sex	2.62	2.52	-2.33	7.57
Age × sex	-0.12	0.07	-0.26	0.02

NB: *** denote 1% significance level.

## Discussion

The two major findings of the study are that, the average healthcare expenditure is higher in male than female and elderly population expend more than younger people. It was also found that no age and sex specific pattern on healthcare expenditure exists in Bangladesh (Figure 1) which also similar with the findings of other studies (Gómez 2002; Ladwig et al. 2000). A number of studies observed that sex and reproductive biology and mortality to male–female differences in the use of health care services, which also reflected in the current study (Mustard et al. 1998; Roos et al. 1987). Considering female, it was seen that healthcare expenditure curve was rising sharply at age range 70–74 and also declining sharply aged over 79 years. However, some other studies also found that most of the elderly female aged 60 years and above received medical service more frequently than male (Áurea et al. 2006; Fernández-Mayoralas et al. 2000; Mutran and Ferraro 1988). Furthermore, policy makers in many countries faces the challenge of this overpressure that elderly populations utilize more health care thus increase in health care expenditure which also confront in this study (Barer et al. 1989).

The results of this study are consistent with findings from a range of health care systems describing higher expenditures for medicine cost and diagnostic care for male than female. Considering the different types of sex specific healthcare services (Table 3) medicine cost is significantly higher in male than female. In childhood period, health care expenditure for male is slightly higher than female which showed the similar findings of other study in Bangladesh (Sarker et al. 2013) and this paper addressed some inequality in case of medicine cost that it provided less frequent in male than female.

This study examined more closely the influence of healthcare expenditures in Bangladesh which help for policy makers plotting health service utilization and healthcare expenditure patterns against age and sex. Nevertheless, there are two important changeable things need to recognized for health policy makers, *firstly,* the age and sex-specific utilization patterns among different age groups over time and *secondly*, the life expectancy of the population which can provide accurate projections of future healthcare expenditure of the country. However, these results provide an experimental framework for the continuing inquiry of equity to provide equal care for both male and female at different stages of human life. Future research needed in this area to provide the effect of age and sex on healthcare demand in Bangladesh.

The study has some limitations. There may be some recall bias as data were collected after receiving the health services. Furthermore, this survey data (HIES-2010) mentions nothing about proxy interviews; in cases where children and elderly people were unable to respond to the interviews, some proxy respondents may have been interviewed. However, we do not have the full access of any datasets of HIES-2000 or 2005, so we were not able to measure inequality across age groups and gender.
